# Consequences of Haemorrhagic Smolt Syndrome (HSS) for the Immune Status of Atlantic salmon (*Salmo salar* L.) (Case Study)

**DOI:** 10.3390/biology9010001

**Published:** 2019-12-19

**Authors:** Aleksei Krasnov, Ingunn Sommerset, Tina Søfteland, Sergey Afanasyev, Preben Boysen, Hege Lund

**Affiliations:** 1 Nofima AS, Norwegian Institute of Food, Fisheries & Aquaculture Research, P.O. Box 5010, 1432 Ås, Norway; Aleksei.Krasnov@nofima.no; 2 Norwegian National Veterinary Institute, Thormøhlensgate 53 C, N-5006 Bergen, Norway; Ingunn.Sommerset@vetinst.no; 3 MSD Animal Health, Thormøhlensgate 55, N-5008 Bergen, Norway; Tina.Softeland@merck.com; 4 Sechenov Institute of Evolutionary Physiology and Biochemistry, M. Toreza Av. 44, Saint Petersburg 194223, Russia; Afanserg@mail.ru; 5 Norwegian University of Life Sciences, Faculty of Veterinary Medicine, P.O. Box 369 Sentrum, 0102 Oslo, Norway; Preben.Boysen@nmbu.no

**Keywords:** Atlantic salmon, haemorrhagic smolt syndrome, microarray, IgM, clonotypes

## Abstract

Haemorrhagic smolt syndrome (HSS) is a disorder of unknown aetiology causing losses in the fresh water phase of Atlantic salmon farming. Normally, the mortality is limited and symptoms disappear upon seawater exposure. In this case study, classical HSS pathology with internal organ haemorrhages and nephrocalcinosis was diagnosed, and the losses were substantial. Microarray analyses of head kidney revealed association between HSS and enhanced expression of stress genes and proteins reducing bioavailability of iron, heme, and retinol. In parallel, suppression of multiple metabolic pathways was observed. Up-regulation of genes encoding acute phase proteins, complement, and lectins indicated mild inflammation but without characteristic features of viral or bacterial infections. Microarray analyses highlighted several members of tumor necrosis factor receptor superfamily that may control development of B-cell immunity. Examination of IgM at the mRNA and protein levels showed the impact of HSS on vaccine responses. In fish without HSS symptoms (non-HSS), titres of vaccine specific antibodies to A-layer of *Aeromonas salmonicida* subsp. *salmonicida* and *Moritella viscosa* and antibodies binding to DNP-keyhole limpet hemocyanin (DNP-KLH), which are presumably polyreactive, were respectively four- and 14-fold higher than in HSS-diseased fish. Parallel sequencing of variable regions of immunoglobulin Mrevealed a larger size of most abundant clonotypes shared by multiple individuals in the non-HSS group. The results of the current case study indicated that, in addition to direct damage, HSS suppresses humoral immune responses including the production of specific and polyreactive antibodies.

## 1. Introduction

Haemorrhagic smolt syndrome (HSS) was first detected in Scotland in 1996 [1] and today fish with the clinical signs of HSS are repeatedly found in Norway [2]. Salmon suffering from HSS are characterized by pale gills and yellow liver, bleeding at the fin bases, low haematocrit, multiple haemorrhage in organs and endothelial tissue, and renal pathology. Loss of equilibrium and lethargy is observed in some affected individuals. Despite severe symptoms, mortality caused by HSS is usually low and HSS does not affect the growth of fish. The disease has been linked to osmoregulatory challenges associated with the process of smoltification and has commonly been observed in the late phase of the freshwater period. To this end, no association between HSS and infectious agents has been shown, and diagnostic tests have not detected any known pathogens. Nylund and co-workers found evidence of two types of virus-like particles in different tissues and cell types by electron microscopy [2]. However, intraperitoneal injection of organ homogenates from HSS-sufferers followed by rearing of fish at different temperatures for long periods did not result in transmission of HSS. Possible aetiologies of the syndrome have been discussed, including toxicity, hypersensitivity, autoimmunity, and endocrine disorders.

The present study was performed as part of an investigation done by MSD Animal Health to monitor vaccinated fish with poor performance associated with a diagnosis of chronic, sub-acute HSS. Fish had also been subjected to a period of low fresh water temperatures. Transcriptome analyses and high-throughput sequencing of the variable region of immunoglobulin M (IgM) (Ig-seq) were performed in the head kidney from HSS-affected salmon and non-affected, apparently healthy controls (non-HSS) from the same tank. Blood serum from the same individuals was analysed for the presence of vaccine-specific antibodies and antibodies binding to DNP-keyhole limpet hemocyanin (KLH), a model antigen consisting of a hapten-carrier complex, which is presumably polyreactive in nature. The results reported herein shed light on the molecular mechanisms of renal pathology associated with HSS and are in line with the hypothesis of a non-infectious origin of the syndrome. The study indicated candidate genes that may reflect the status of B-cell immunity in fish and demonstrated the potential utility of polyreactive antibodies for the assessment of immune status of Atlantic salmon.

## 2. Material and Methods

### 2.1. Fish

Atlantic salmon (*Salmo salar*) were vaccinated with a commercially available inactivated, multivalent injection vaccine (Aquavac PD7 vet.^®^, MSD Animal Health, Bergen Norway) at an average size of 122 g in a flow-through freshwater facility in Norway. Approximately one month after vaccination, fish were diagnosed by autopsy and histopathology by local veterinary fish health personnel to be suffering from classical HSS without elevated mortality. Two months later, the fish were subjected to a temperature drop followed by a period with cold water temperatures (0.5–2 °C) and the mortality increased. Sub-acute to chronic phase of HSS and nephrocalcinosis was diagnosed. No other diagnosis was found by clinical examination and histopathology, and repeated PCR-testing for Piscine orthoreovirus (PRV) and infectious pancreatic necrosis virus (IPNV), due to historical outbreaks at the farm, was negative. Over a subsequent six weeks period, the accumulated mortality in the affected tanks varied from 5–30%. At 18–19 weeks after vaccination, head kidney and blood serum from nine fish with HSS pathology (hereafter HSS) and seven apparently healthy controls (hereafter non-HSS) were collected from the same tank. All samples were taken prior to sea water transfer. The head kidney samples were stored in RNA*later* (ThermoFisher). The serum samples were prepared by spinning whole blood collected from the caudal vein after approximately 2 h of clotting at 2–8 °C.

### 2.2. Microarray

Nofima’s Atlantic salmon genome-wide DNA oligonucleotide microarray Salgeno contains 44k probes to protein coding genes. The genes were annotated by GO, KEGG, Interpo, and custom vocabulary using Nofima’s bioinformatics package STARS [3]. RNA was extracted with PureLink RNA Mini Kit (Thermo Fisher Scientific, Waltham, MA, USA) according to the manufacturer’s instructions, and RNA was stored at −80 °C until use. Samples included for microarray analysis were head kidney RNA from HSS sufferers (*n* = 6) and non-HSS (*n* = 6). Microarrays were manufactured by Agilent Technologies, and the reagents and equipment were purchased from the same provider. RNA amplification and labelling were performed with a one-color quick amp labeling kit, and a gene expression hybridization kit was used for fragmentation of labelled RNA. Total RNA input for each reaction was 500 ng. After overnight hybridization in an oven (17 h, 65 °C, rotation speed 0.01 g), arrays were washed with Gene Expression Wash Buffers 1 and 2 and scanned with an Agilent scanner. Subsequent data analyses were performed with STARS. Global normalization was performed by equalizing the mean intensities of all microarrays. Next, the individual values for each feature were divided by the mean value of all samples producing expression ratios (ER). The log2-ER were calculated and normalized with the locally-weighted nonlinear regression (Lowess). Differentially expressed genes (DEG) were selected by the following criteria: expression ratio >1.75-fold and *P* < 0.05. Enrichment of functional categories of GO and KEGG pathways was assessed by comparison of counts in the list of DEG and the microarray platform (*P* < 0.05, Yates’ corrected chi-square test).

### 2.3. Ig-seq

Sequencing of IgM variable regions in the head kidney (HSS = 9 and non-HSS = 7) and subsequent data analyses were carried out basically as described in [4]. All reagents, unless indicated otherwise, were from Thermo Fisher Scientific Invitrogen. In brief, cDNA was synthesized using Super Script II and IGMR1 primer to the 5′-end of constant region of Atlantic salmon IgM heavy chain (TAAAGAGACGGGTGCTGCAG), and purified with Amicon YM30 membrane filters (Merck Millipore, Burlington, MA, USA). Purified cDNA was tailed with dC at the 5′-end using terminal deoxynucleotidyl transferase (TdT). Three consecutive PCR amplifications were performed in a 50 µL volume with Platinum Taq DNA polymerase. The first PCR (30–32 cycles) used IgM-specific IGMR2 (TGGTTTGGACAGCAGGGTAC), Invitrogen abridged amplification primer AAP (GGCCACGCGTCGACTAGTACGGGIIGGGIIGGGIIG), and 5 µL of tailing reaction product. The second PCR (10 cycles) amplified 5 µL of PCR1 product with primers containing sequences of Invitrogen abridged universal amplification primer (AUAP) and IgM (underlined) linked to Illumina adaptors: NEXU (GTCTCGTGGGCTCGGAGATGTGTATAAGAGACAGGGCCACGCGTCGACTAGTAC) and NIGM (TCGTCGGCAGCGTCAGATGTGTATAAGAGACAGGGAACAAAGTCGGAGCAGTTGATGA). Finally, the third PCR was carried out with 5 µL of PCR2 product and index primers from Illumina Nextera Index kit. Sequencing with Illumina MiSeq Reagent Kit v2 (read one, 200 cycles from the 3’-end) was performed according to manufacturer’s instructions. After trimming the primer, sequences were filtered (Illumina q > 20) and the structural elements of IgM-VR were identified using Smith–Waterman algorithm for J and blastn for V segments. Sequences were translated in three frames and complementarity determining region 3 (CDR3) were determined by guidelines of IMGT [5]. Clonotypes were assigned by combinations of J and V genes and unique amino acid sequences of CDR3. In each sample, clonotypes represented with at least two transcripts were used for further analyses. Clonotypes were denoted as unique if transcripts were detected in a single fish or shared if transcripts were found in at least two individuals with a transcript frequency of at least 1 per 10,000. The hundred most abundant clonotypes were selected in each individual and cumulative frequencies of unique and shared clonotypes were calculated. Statistical analysis was performed by the GraphPad Prism 7 software (Mann–Whitney test, *P* ˂ 0.05).

### 2.4. Direct ELISA for Total IgM and Blood Cell Count

Total IgM was measured in serum (HSS = 9 and non-HSS = 7) by a direct ELISA based on the method published by Magnadottir [6] and performed by Skretting AS using a commercial trout/salmon IgM antibody (Aquatic Diagnostics Ltd., University of Stirling, Scotland, UK).

The number of viable nucleated cells in Atlantic salmon (HSS = 9 and non-HSS = 7) whole blood was calculated by the use of a NucleoCounter^®^ (ChemoMetec, Allerød, Denmark.), following the instructions of the instrument. Viable nucleated cells in blood were calculated as number of cells per mL of full blood.

### 2.5. Bead Coupling and Multiplex Immunoassay

For detection of specific antibodies after vaccination, the A-layer protein from *Aeromonas salmonicida* subsp. *salmonicida* [7], (in-house produced), and whole cell sonicate from *Moritella viscosa* type strain 1016/96 (kindly provided by Liv Jorun Reitan, Norwegian Veterinary Institute) were included in the multiplex assay. For detection of polyreactive antibodies, the hapten-carrier complexes DNP-keyhole limpet hemocyanin (DNP-KLH) and DNP-bovine serum albumin (BSA) (both Calbiochem, Merck, Darmstadt, Germany), and DNP-lipopolysaccharide (LPS) (from *Escherichia coli* 026:B6) and DNP-ovalbumin (OVAL) (both Biosearch technologies, Novato, CA, USA) were included. Non-haptenated carrier proteins were BSA (Calbiochem), and KLH, LPS and OVAL (Sigma-Aldrich, St. Louis, MI, USA). Antigens were coupled to distinct MagPlex^®^-C Microspheres (Luminex Corp., Austin, TX, USA) of different bead regions according to the manufacturer’s protocol using the Bio-Plex amine coupling kit (Bio-Rad, Hercules, CA, USA). Haptenated and non-haptenated antigens were used at an amount of 10 µg per 1× scale coupling reaction, and A-layer protein and *M. viscosa* sonicate at an amount of 12 µg and 7 µg, respectively. The immunoassay was performed as previously described [8]. Briefly, beads were diluted in assay buffer (PBS with 0.5 % BSA and 0.05 % azide), and 5000 beads per region were added to each well. Beads were washed three times with assay buffer (30 s in the dark and on a shaker at 800 rpm), then kept for 120 s in a Bio-Plex handheld magnetic washer before the supernatant was poured off. Serum samples (HSS = 9 and non-HSS = 7) were diluted 1:200 in assay buffer and added in duplicates on the plate. The plate was incubated for 30 min at room temperature (RT) in the dark and on a shaker at 800 rpm. All subsequent incubation and washing steps were performed similarly. Following incubation and washing, anti Salmonid-IgH monoclonal antibody was added to the wells (1:400, clone IPA5F12, Cedarlane, Burlington, ON, Canada). After incubation and washing, biotinylated goat Anti-Mouse IgG2a antibody (1:1000, Southern Biotechnology Association, Birmingham, AL, USA) was added in each well, and finally, after incubation and washing, Streptavidin-PE (1:50, Invitrogen) was applied. Plates were analyzed using a Bio-Plex 200 in combination with Bio-Plex Manager 6.1 software (Bio-Rad). Each bead was classified by its signature fluorescent pattern and then analyzed for the median fluorescent intensity (MFI) of the reporter molecule. Statistical analysis was performed by the GraphPad Prism 7 software (Mann–Whitney test, *P* ˂ 0.05).

### 2.6. Ethical Statement

The cases reported in the present study were from a natural outbreak of hemorrhagic smolt syndrome in a commercial Atlantic salmon farm. All animal handling was performed in compliance with the regulatory requirements by The Norwegian Food Safety Authority and according to guidelines of Good Laboratory Practice (EU Council Directive 2004/10/EC). 

## 3. Results

### 3.1. Microarray

**Figure 1:**
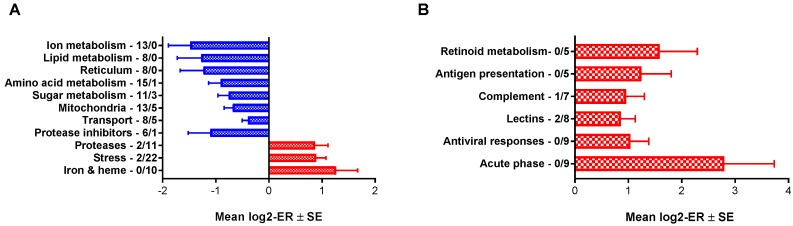
Functional groups of genes with co-ordinated expression changes in HK. (**A**) Stress and metabolism. (**B**) Immune response. Data are mean log2 (ER) ± SE (ER: HSS to non-HSS expression ratio). Positive (red columns) and negative (blue columns) values mean respectively higher and lower levels in salmon with HSS. Numbers of down- and up-regulated genes are indicated.

Expression differences between HSS-affected and healthy control salmon were shown by 542 genes, of which 327 genes (60.3%) had lower expression in HSS fish. Overall, salmon diagnosed with HSS exhibited suppression of several metabolic pathways, with the exception of metabolism of iron and heme in combination with stimulation of protein degradation and stress (Figure 1A).

Up-regulation of genes encoding iron (*fth1b*) and heme (*hp*) binding proteins, heme degrading enzyme *ho*, and the master regulator of iron metabolism *hepc1* are likely to be associated with the observed haemorrhage (Table 1). A suite of chaperones (*hsp90a*, *hsc70*, *grp78*, and *grp94*), normally associated with stressed tissue conditions, and genes that consistently respond to various disturbances in Atlantic salmon (*cebp*, *gadd4*5, *junb*, and *hif*—data from Nofima’s microarray database) were upregulated in HSS sufferers. Genes encoding protein degrading enzymes were stimulated in parallel with down-regulation of protease inhibitors. Table 1 also includes genes whose down-regulation might cause functional disorder in the kidney of salmon with HSS, including the water channel *aqp8b* and *Na+/K+ ATPases* which maintain the osmotic balance. Decreased transcription was shown by a large group of genes encoding transporters excreting diverse anions including phosphorus (*slc34*), carnitine (*slc22a4*), mono-(*slc5a8*), and dicoarboxylates (*slc13a3* and *SLC22a6*). *Mrp* and *slc22a7* transport multiple endogenous metabolites and exogenous anions including toxicants and drugs. A particularly strongly upregulated gene was *s100-a11*, which encodes a calcium binding protein with broad biological functions [9] including some associations with low-grade inflammation [10].

Genes from several functional groups within inflammation and innate immunity were up-regulated in HSS-affected salmon, especially acute phase proteins, lectins, and complement (Figure 1B, Table 2). Several of these are soluble components which bind to bacteria (antibacterial peptide *camp*, multiple scavengers, lectins, and lectin receptors). The greatest increase of expression was shown by two *c1q-like* genes with unknown roles. Several other genes with broad inflammatory functions were down-regulated, including *chia* (chitinase) [11], *mpo* (myeloperoxidase) [12], and *il8* [13]. Expression differences were found in several genes associated with adaptive immune activation. Most prominently, a near 20-fold higher expression of tumor necrosis factor receptor superfamily member 11b (*tnfrsf11b*) was seen in the HSS group. Its mammalian orthologue, also known as osteoprotegerin, is a soluble decoy receptor acting as a “molecular brake” on RANK/RANKL signalling, which is central in bone modelling and remodelling as well as B-cell development and function, with possible consequences for efficient antibody production [14,15]. Concurrently, several other factors associated with B-cell function were downregulated in HSS sufferers: *ebf1a*, which is crucial in B-cell development and whose disruption led to selective B-cell deficiency in zebrafish [16], *tnfrsf13b* (TACI), a central receptor in the APRIL/BAFF pathway suggested to have a B1-cell specific function, and *tnfrsf14* (HVEM), an inhibitory receptor expressed on B- as well as T-cells [17]. Several genes involved in metabolism of retinol showed higher expression in HSS. High expression of retinol-binding proteins may reduce bioavailability of retinol, which is essential for differentiation and functions of B- and T-cells [18]. Five genes involved in antigen presentation showed a higher expression in HSS-affected fish. Genes belonging to the MHCII system (*hla-dpa* and *hla-dpg*) were up-regulated concurrently with T-helper cell associated markers *cd4*, *cd28*, and *cd274*. Seen together, genes of the innate immune system were both up- and down-regulated, while a series of expression changes could indicate dampening of adaptive immunity, with particular bias towards B-cell suppression while the presence of T helper cells appeared unaffected or increased. Complete microarray data (Table S1), DEG (Table S2), and enriched terms (Table S3) are available in the Supplementary Materials.

### 3.2. Ig-seq, Blood Cell Count, and Serological Immunoassays

**Figure 2:**
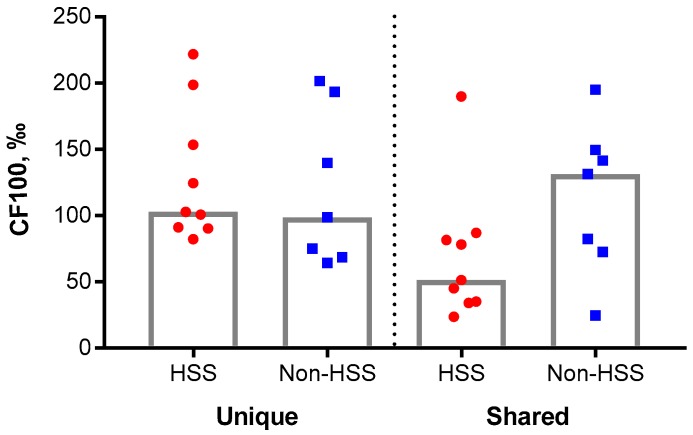
Deep sequencing of the variable regions of IgM. Cumulative frequencies (CF) of transcripts from the hundred most abundant unique and shared clonotypes in HSS-affected (red circles, *n* = 9) and non-HSS-affected (blue squares, *n* = 7). Median value of each group is illustrated by a grey column.

HSS-affected and healthy control salmon were compared by the hundred most abundant immunoglobulin clonotypes (leaders), which presumably included those that responded to immunization and/or to other unknown antigens. Cumulative frequencies of transcripts (CF) were calculated for clonotypes detected in one (unique) or several (shared) individuals. CF of shared clonotypes were higher, however not significantly, in non-HSS compared to HSS-affected salmon, while frequencies of unique clonotypes were equal in both groups (Figure 2).

**Figure 3:**
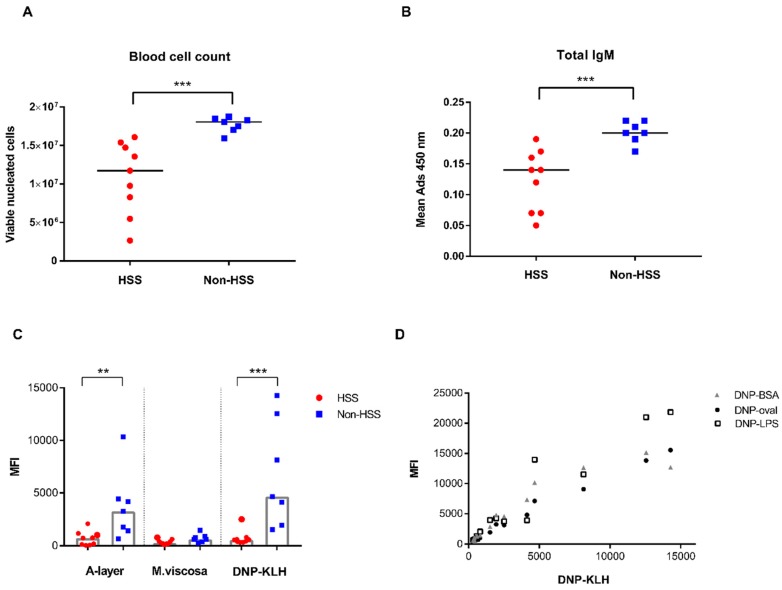
Blood cell count and immunoassays. (**A**) Number of viable nucleated cells per ml of full blood in Atlantic salmon, calculated by NucleoCounter^®^. (**B**) Measured optical density of total IgM in serum at a mean adsorbance of 450 nm. (**C**) Salmon IgM antibodies recognizing antigens delivered by vaccination and a model antigen DNP-KLH. Median value of each group is illustrated by a grey column. (**A**–**C**). All individual data of HSS-affected (red circles, *n* = 9) and non-HSS-affected fish (blue squares, *n* = 7) are shown. Statistical significant between HSS and non-HSS are indicated with ** (*P* < 0.01) and *** (*P* < 0.001). (**D**) Correlation of antibody titres to DNP-KLH and three other DNP-haptenated antigens determined in multiplex assays. MFI = median fluorescent intensity.

A significant decrease in the number of viable nucleated cells was found in the HSS-affected fish (Figure 3A). The same group also showed a significant decrease in measured optical density of total IgM in serum (Figure 3B). The bead-based multiplex immunoassay measured antibodies to antigens delivered with vaccine and antibodies binding to a haptenated model antigen, DNP-KLH. The levels of antibodies binding to the A-layer protein of *A. salmonicida* subsp. *Salmonicida*, but not to the whole cell sonicate of *M. viscosa*, were significantly lower in HSS-affected fish (*P* ˂ 0.05, Figure 3C). Levels of antibodies binding to DNP-KLH were also significantly lower in the HSS group (*P* ˂ 0.001). The differences in antibody titres between HSS and non-HSS were much greater for anti-DNP-KLH and polyreactive antibodies than specific antibodies directed towards antigens delivered with the vaccine. Antibody titres recognizing DNP-KLH were found to be highly correlated with antibody titres towards three other DNP-haptenated model antigens (BSA, ovalbumin, and LPS) in non-HSS fish (Pearson r = 0.94–96, Figure 3D). Levels of antibodies to the carrier proteins KLH, BSA, and ovalbumin alone were low, except for antibodies to LPS, which were comparable by magnitude to the antibody levels to the A-layer protein of *A. salmonicida* subsp. *salmonicida* (data not shown).

## 4. Discussion

The aetiology of haemorrhagic smolt syndrome remains unknown. At present it is impossible to induce this syndrome under experimental conditions and HSS can therefore be studied only on field material. The pathological changes associated with HSS were described by Nylund and co-authors [2]. The main focus of the present study was the effect of HSS on the immune status of salmon. We examined a case where the losses were high, probably as a consequence of combined HSS and a stressful drop in temperature. However, the sufferers and healthy fish were kept under equal conditions and extensive investigation revealed a severe form of HSS as the only disease. 

Transcriptome analyses of the head kidney supported the hypothesis of a non-infectious origin of HSS. Nofima’s database stores results of microarray analyses performed in numerous studies of Atlantic salmon infected with different viral and bacterial pathogens, and meta-analyses of these data have revealed diagnostic transcription signatures. Viral infections commonly activate a large group of genes involved in innate antiviral immunity termed VRG—virus responsive genes [19]. VRG can be induced with other agents, for example with bacterial DNA in the form of plasmids [20], and in theory this may complicate the conclusions. However, lack of VRG stimulation points to absence of a pathogenic virus in an active state. Although salmon genes specifically activated with bacteria have not been identified, infections may be recognized by the magnitude and character of immune responses. Bacterial pathogens usually induce recruitment and activation of leukocytes manifested by enhanced levels of transcripts encoding damaging effectors, such as the components of the oxidative burst complex and matrix metalloproteinases. Such changes were not observed in the present study. A similar response could be expected if the condition was associated with strong alterations in microbiota and dysbiosis-induced inflammation, as observed in zebrafish [21]. Mild inflammatory responses in salmon with HSS most likely developed as consequence of tissue damage, haemorrhage and stress responses. Genes expected to have an increased expression during bacterial infection and neutrophil activation, such as IL-8 and myeloperoxidase, were down-regulated. Expression profiles that reveal infectious diseases are commonly observed in the tissues which harbour pathogens, and therefore one cannot rule out the possibility that the focus of infection in the present study was not located in the head kidney. However, given that HSS is a systemic disorder affecting primarily endothelial tissue and that the kidney is one of the most affected organs, this assumption is unlikely. Bacteria can complicate the disease but are most likely not the primary cause. 

High titres of natural or heterologous antibodies have been reported in multiple fish species [22,23,24], and our group recently demonstrated the increase of non-vaccine specific antibodies after vaccination of Atlantic salmon [8]. In the former and current study, proteins linked to DNP were tested since it has been shown that sera of bony fish preferably interact with hapten–carrier model antigens [24]. A strong correlation of titres against four haptenated antigens suggested that the assays detected the same anti-DNP antibodies, whereas close to baseline signals to carrier proteins suggested that their contribution was negligible. To denote antibodies revealed by binding to model antigens, we suggest the term polyreactive antibodies, meaning antibodies that recognize antigens that fish have never been exposed to and which presumably have a polyreactive nature. In the present material, a drastic reduction of both specific antibodies directed against components of the vaccine and of polyreactive antibodies binding to DNP-KLH was observed in HSS-affected fish. Anti-DNP-KLH antibodies of Atlantic salmon possibly recognising multiple antigens may be involved in protection against infections. Fish challenged with PRV showed higher resistance to salmonid alphavirus (SAV) [25] and plasma from several PRV-infected individuals neutralized SAV in vitro in a heat sensitive manner [26]. Our group has observed a polyreactive nature of antibodies from Atlantic salmon infected with PRV [27] and SAV (unpublished results). The occurrence of polyreactive heterologous antibodies was shown in carp (*Cyprinus carpio*) that survived herpesvirus infection, where sera of infected, but not naïve, fish interacted with heterologous viruses [22]. In parallel to the reduction of circulating antibodies in HSS fish, a lower cumulative frequency of transcripts from shared clonotypes was seen in this group compared to the non-HSS group, indicating a lower degree of clonotype expansion. In contrast, the higher frequency of shared clonotypes observed in apparently healthy fish suggests an expansion of clonotypes shared by several individuals, most likely as a response to the vaccination in this group.

Gene expression profiles suggested that suppressed immune responses in HSS fish were most likely not related to deficiency of antigen presentation and T helper cells, as expression of MHCII components and *cd4* and other T-cell markers was higher in salmon with HSS. A small number of genes with potential impact on B-cell immunity showed difference between HSS and non-HSS; elevated retinol-binding proteins, as well as osteoprotegerin are soluble factors that could potentially disrupt B-cell function, and lowered TACI, *ebf1*, and possibly other TNFR receptor superfamily genes could disturb normal B-cell function. Altogether, expression of these genes may explain the severely dampened antibody responses observed in HSS sufferers. However, as the pathology of HSS is indicative of increased endothelial tissue permeability with widespread haemorrhages, protein loss caused with the generalized bleeding might contribute to the lower total IgM and antibody levels in HSS-affected fish. 

Apart from inflammation, the renal transcriptome of salmon with HSS was characterized by activation of heme and iron sequestration combined with cellular stress. Gene expression suggests neutralization of bioavailable heme and iron as key protective measures aimed at counteracting damage from haemorrhage. Free heme and iron are potent catalysers of free radicals, which should be immediately removed [28]. Down-regulation of multiple transporters might impair the renal functions. Change in osmoregulation is essential for adaptation of farmed salmon to seawater, and isoforms of the a1 chain of Na+/K+ ATPase are used as markers of smolt quality and readiness to the marine environment [29,30]. In the present study, down-regulation of two other chains of this complex (*b1a* and *a2* encoded with respectively two and three genes) was found, which are commonly up-regulated in the head kidney of Atlantic salmon during smoltification [31].

The results of the present study indicate that HSS, in addition to direct damage, leads to suppressed humoral immune responses in Atlantic salmon. Though recovery from HSS usually takes place after transfer to seawater, long-term consequences may manifest as a weakened immune defence. Whether antibodies of a polyreactive nature protect against pathogens or not, they are markedly stimulated with infections and vaccination. Striking differences between HSS-affected and healthy control fish suggest that polyreactive antibodies can be a useful marker of fish health status and responses to vaccination.

## Figures and Tables

**Table 1:** Differentially expressed genes: stress and metabolism. Data are HSS/non-HSS fold changes, and all differences between the groups are significant. Gene symbols and numbers of paralogous genes are in parentheses.

	Fold Difference HSS/non-HSS
***Stress and iron metabolism***	
Ferritin, heavy polypeptide 1b (*fth1b*, 4 genes)	1.8–2.1
Haptoglobin (*hp*, 3 genes)	6.3–6.7
Heme oxygenase (*ho*, 4 genes)	1.8–4.7
Hepcidin-1 (*hepc1*)	3.5
Glucose-regulated protein 78 kDa (*grp78*)	2.2
Glucose-regulated protein 94 kDa (*grp94*)	2.0
Heat shock cognate 70 (*hsc70*, 7 genes)	2.0–2.5
Heat shock protein 90, alpha (*hsp90a*)	1.8
C/EBP beta (2 genes)	2.1–2.2
C/EBP delta	2.6
GADD45 beta (2 genes)	1.9–2.1
Hypoxia-inducible factor 1a (*hif1a*, 2 genes)	2.2–2.4
Jun B-1	1.9
Cathepsin L1 (*catl1*, 2 genes)	2.6–2.8
Cathepsin S (*cats*)	2.3
Cathepsin Z (*catz*)	1.8
***Ion metabolism and transport***	
S100-A11 (2 genes)	7.6–14.4
Na+/K+ ATPase, b1a (*at1b1*, 2 genes)	−(1.9–2.7)
Na+/K+ ATPase a2 (*at1a2*, 3 genes)	1.8–2.3
Aquaporin 8b (*aqp8b*, OrthoDB)	−2.9
Multidrug resistance-associated protein (*mrp*)	−2.9
Solute carrier family 5 member 8a (*slc5a8*)	−2.0
Solute carrier family 13 member 3 (*slc13a3*)	−2.7
Solute carrier family 22 member 4 (*slc22a4*)	−2.6
Solute carrier family 22 member 6-A (*slc22a6*)	−1.8
Solute carrier family 22 member 7 (*slc22a7*)	−2.9
Solute carrier family 34 (*slc34*)	−2.0

**Table 2:** Differentially expressed genes: immune genes. Data are HSS/non-HSS fold changes, and all differences between the groups are significant. Gene symbols and numbers of paralogous genes are in parentheses.

Immune Genes	Fold Difference HSS/non-HSS
C-C motif chemokine 13	3.6
C-C motif chemokine 19-4	5.2
Serum amyloid A5 (*saa5*)	6.2
C type lectin receptor A	3.1
Cathelicidin (*camp*)	6.1
C-type lectin 4E (*clec4e*)	6.1
Macrophage receptor MARCO (*mrc1*)	2.5
Mannose receptor	2.6
C1q components b, c (3 genes)	1.9–3.1
C1q-like (2 genes)	15.7–17.4
C1q-like 2 (c1ql2)	5.0
C6	2.1
H-2 class II HC antigen, a chain (*hla-dpa*)	2.8
H-2 class II HC antigen, g chain (*hla-dpg*, 2 genes)	1.9–2.2
CD274	2.4
CD28	3.0
CD4-like	3.4
TNF receptor superfamily member 11B (*tnfrsf11b*)	19.6
Retinol-binding protein 1a, cellular (*ret1a*)	3.0
Retinol-binding protein 4 (*ret4*, 2 features)	3.7–4.0
Chitinase, acidic.3 (*chia*)	−7.5
Myeloperoxidase (*mpo*)	−2.0
IL8	−2.7
Mitogen-activated protein kinase 12b (*mapk12b*)	−3.1
Mitogen-activated protein kinase-activated protein kinase 3 (*mapkapk3*)	2.8
TNF receptor superfamily member 13b (*tnfrsf13b*)	−2.2
Early B-cell factor 1a (*ebf1a*)	−4.0
TNF receptor superfamily member 14 (*tnfrsf14*)	−2.3

## Supplementary Materials

The following are available online at https://www.mdpi.com/2079-7737/9/1/1/s1, Table S1: Microarray results, Table S2: Differentially expressed genes (DEG), Table S3: Enrichment analysis.
